# Biomass and carbon stock models with climatic factors for individual Quercus *mongolica* trees and their allocation patterns

**DOI:** 10.1186/s13021-026-00414-8

**Published:** 2026-02-08

**Authors:** Jun Lu, Lingbo Dong, Hao Zhang

**Affiliations:** 1 Present Address: Institute of Forest Resource Information Techniques, Chinese Academy of Forestry, Beijing, 100091 P. R. China; 2 School of Forestry, Northeast Forestry University, Harbin, 150040 P. R. China

**Keywords:** Mongolian oak, Single tree biomass, Climatic factor, Compatible biomass model, Allocation patterns

## Abstract

As the environmental problems caused by the greenhouse effect become more and more serious, and the forest as the largest carbon pool can effectively slow down the greenhouse effect, it is particularly important to accurately predict the carbon storage of the forest. In order to accurately estimate the biomass and carbon storage of Quercus *mongolica* in Northeast China, the biomass allocation pattern of Q. *mongolica* was analyzed. In this study, data of 175 Q. mongolica trees in Heilongjiang, Jilin, Liaoning and eastern Inner Mongolia were collected, including aboveground organ biomass, DBH, tree height, age and climatic factors, as well as published carbon content data of different organs. In this study, the biomass allocation pattern of individual Q. *mongolica* was analyzed. An additively compatible aboveground biomass and carbon storage model and an algebraically controlled aggregation model were established using nonlinear simultaneous equations. After selecting the aggregate biomass compatibility model, climate factors were added to establish a compatibility model containing climate factors. In addition, the root-stem ratio model was used to construct the underground compatible biomass and carbon storage model. The adjusted R^2^_adj_ values of the final established aboveground components and aboveground total biomass and carbon storage models were between 0.7048 and 0.9618, the total relative error ( TRE ) was within ± 1%, and the average prediction error ( MPE ) was below 10%, which met the modeling accuracy standard. The belowground biomass models showed adjusted R²_adj_ values between 0.7702 and 0.7801, TRE ≤ 1%, and MPE < 15%. This study elucidated the biomass allocation pattern of individual Q. *mongolica*. All the developed models meet the accuracy requirements and can be applied to predict the biomass and carbon storage of Q. *mongolica* in Northeast China. In the compatibility model with climate factors, the accuracy of leaf and branch models has been greatly improved, indicating that the addition of climate factors in the independent model can greatly improve the accuracy of each component model, which can provide a theoretical basis for the establishment of each component model in the compatibility model of other tree species.

## Introduction

With the continuous expansion of the greenhouse effect, carbon emissions have received more and more attention, leading to the rise of research on carbon stocks worldwide. As a major component of terrestrial ecosystems, forests account for 40% of belowground carbon storage and 80% of aboveground carbon storage in these ecosystems [1, 7, 12]. In order to mitigate the greenhouse effect and mitigate global extreme climate change, it is very important to accurately estimate forest carbon stocks. The basis of forest carbon storage is individual tree carbon storage, and the main carrier of individual tree carbon storage is individual tree biomass. At present, most studies use direct or indirect methods to estimate individual tree carbon storage by multiplying tree biomass by the carbon content of different plant components. Although the direct method is accurate, it often involves tree felling and sample weighing, and the cost is high. In contrast, although the indirect method is more cost-effective, it may introduce errors due to inaccurate conversion coefficients, making predictive modeling the best choice [11, 16, 18].

In recent years, the concept of biomass model has attracted more and more attention at home and abroad, and the research on biomass model is also hot all over the world [3, 28, 30, 52]. Due to the feasibility of carbon density measurement, stand-level biomass and carbon storage models have been extensively studied [15, 18, 53]. However, due to the wide distribution of samples and unclear carbon content data, carbon storage models at the individual tree level are rarely established. When establishing a single tree biomass model, it is necessary to consider the distribution of biomass among different organ components. Studying the proportion distribution of biomass of each component in each stand factor, such as age, DBH and tree height, can more intuitively understand the dynamic distribution of biomass proportion with these factors. The distribution of biomass in different components also affects the productivity of individual trees, which in turn affects the spatial distribution of stand-level biomass and carbon storage. Therefore, understanding the biomass allocation patterns of individual tree species is crucial for developing accurate individual tree models.

When the carbon content of each component in the national standard is used to calculate the carbon storage of each component in different data, there is also a compatibility problem in the individual tree or aboveground biomass model obtained by adding the components. At present, most individual tree biomass models are constructed using allometric growth equations [4, 6, 34, 38]. In previous studies, individual tree biomass models tend to select the aggregate model to estimate the total biomass of trees by fitting individual components such as stem, bark, branches and leaves. However, this method will lead to the situation that the sum of biomass of individual components is not equal to the total biomass. Establishing a compatible and additive single tree biomass model can effectively solve this problem [43, 48, 56].

Compatible additive biomass models can be divided into two categories according to their construction principles decomposition additive models and aggregation additive models [33, 38, 52]. The decomposition method establishes a compatible model by hierarchically assigning independent individual tree or aboveground total models to each component. In contrast, the aggregation method enhances additivity by limiting the total biomass to be equal to the sum of all components, without requiring an independent whole tree model. The establishment of decomposition and aggregation compatible models expanded the method selection of individual tree biomass and carbon storage estimation system. Both aggregated and decomposed compatible models need to establish nonlinear simultaneous equations. For the parameter estimation of nonlinear equations, the main methods include nonlinear seemingly unrelated regression (NSUR) and ordinary least squares (OLS). Zhao et al.'s research shows that when there is a correlation between the errors of different biomass components, the seemingly unrelated regression method is superior to the ordinary least squares method in model performance [54].

Climatic factors can significantly affect the growth of individual trees. Studying the effects of various climatic factors on tree growth can improve productivity and provide a theoretical basis for forest managers to make scientific decisions [2, 8, 24]. Zeng et al.'s improved the accuracy of the model by using the annual average temperature and annual precipitation as parameters in the compatible model, and also determined the main climatic effects on tree growth [50]. Similarly, Zou *et al. ’s* proved that the mean coldest month temperature (MCMT) and mean annual precipitation (MAP) were the key climatic determinants of secondary forests, and the accuracy of the model was greatly improved after adding them [57]. In the past, the introduction of climate models was often by adding the total amount independent model or the aboveground biomass independent model, and there were few articles considering the addition of climate factors from the perspective of each component. Trying to introduce climate factors into the model can not only improve the accuracy of the independent model, but also whether it can be added to the compatibility model. Whether it can improve the overall accuracy of compatibility is also a question worth exploring.

Q. *mongolica* is a deciduous tree belonging to Quercus of Fagaceae. It is native to the south of Daxing ‘an Mountains in northeastern China. Its distribution range throughout the Northeast, Northwest, North China and North Korea, Japan, Mongolia, Russia and other places. This kind of tree species with economic value has the characteristics of durability and corrosion resistance, and is widely used in commercial forestry. In addition, its branches can be used as fuelwood with high energy consumption, while its acorns and leaves have industrial and feed uses. Ecologically, Q. *mongolica* plays a vital role in soil conservation and water conservation.

Although there are many individual tree biomass models for different species around the world, the research on Q.*mongolica* is still limited in depth and methodological diversity. Adding climate factors to each component model in the compatibility model to re-fit the model may also be a promising method to improve the accuracy of the model. Therefore, the purpose of this study is to study the relationship between the distribution pattern of Q.*mongolica* biomass components and stand factors and climatic factors. The independent, decomposed and aggregated models of aboveground and underground biomass and carbon storage were established and analyzed by univariate and bivariate methods. After screening, the aggregate compatibility basic model was selected, and a climate-enhanced compatibility model was established to estimate biomass and carbon storage.

## Materials and methods

### Overview of the study area

**Fig. 1:**
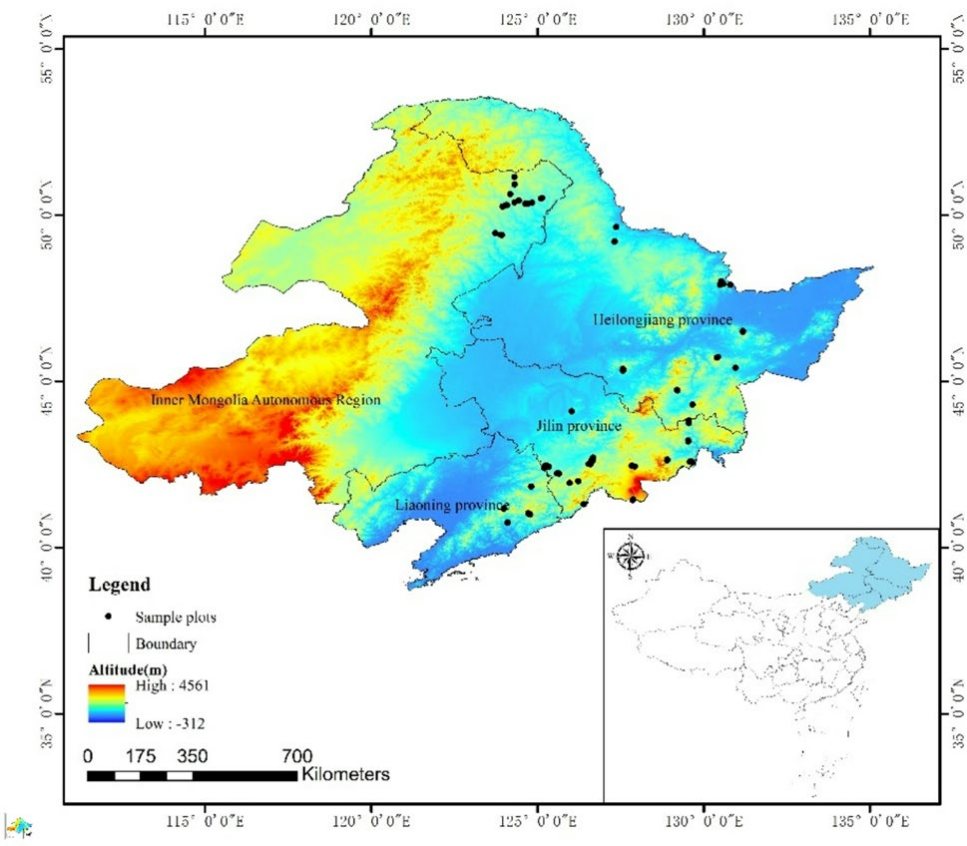
Study area map of Northeast China

The study area is located in Heilongjiang Province, Liaoning Province, Jilin Province and the eastern part of Inner Mongolia Autonomous Region (38°42′~53°33′N, 115°31′~135°34′E). There are many plains and mountains in the area. The mountains include Changbai Mountains and Xing ‘an Mountains. The plain mainly includes the Sanjiang Plain in the east, the Songnen Plain in the middle and the Liaohe Plain in the south. The soil is mainly black soil, and the land is fertile. The region is a temperate monsoon climate, the annual temperature difference is large, and the distribution of forest resources is concentrated. Due to the high forest coverage in Northeast China, the melting time of ice and snow is elongated, which is conducive to the growth of trees. The map of the study area is shown in Fig. 1.

### Data sources

The data used in this study are from three provinces in Northeast China - Heilongjiang, Liaoning and Jilin - and the eastern part of Inner Mongolia Autonomous Region. A total of 175 sample trees were harvested, and 160 trees were selected according to the diameter grades of 2,4,6,8,12,16,20,24,28 and 32 cm (average 16 trees per class). The remaining 15 trees were randomly sampled. Diameter at breast height (DBH) was measured before logging, and tree height and age were recorded after logging. The sample tree was selected according to the tree height and DBH criteria, while ensuring that it was representative and had no bifurcation or top fracture.

**Fig. 2:**
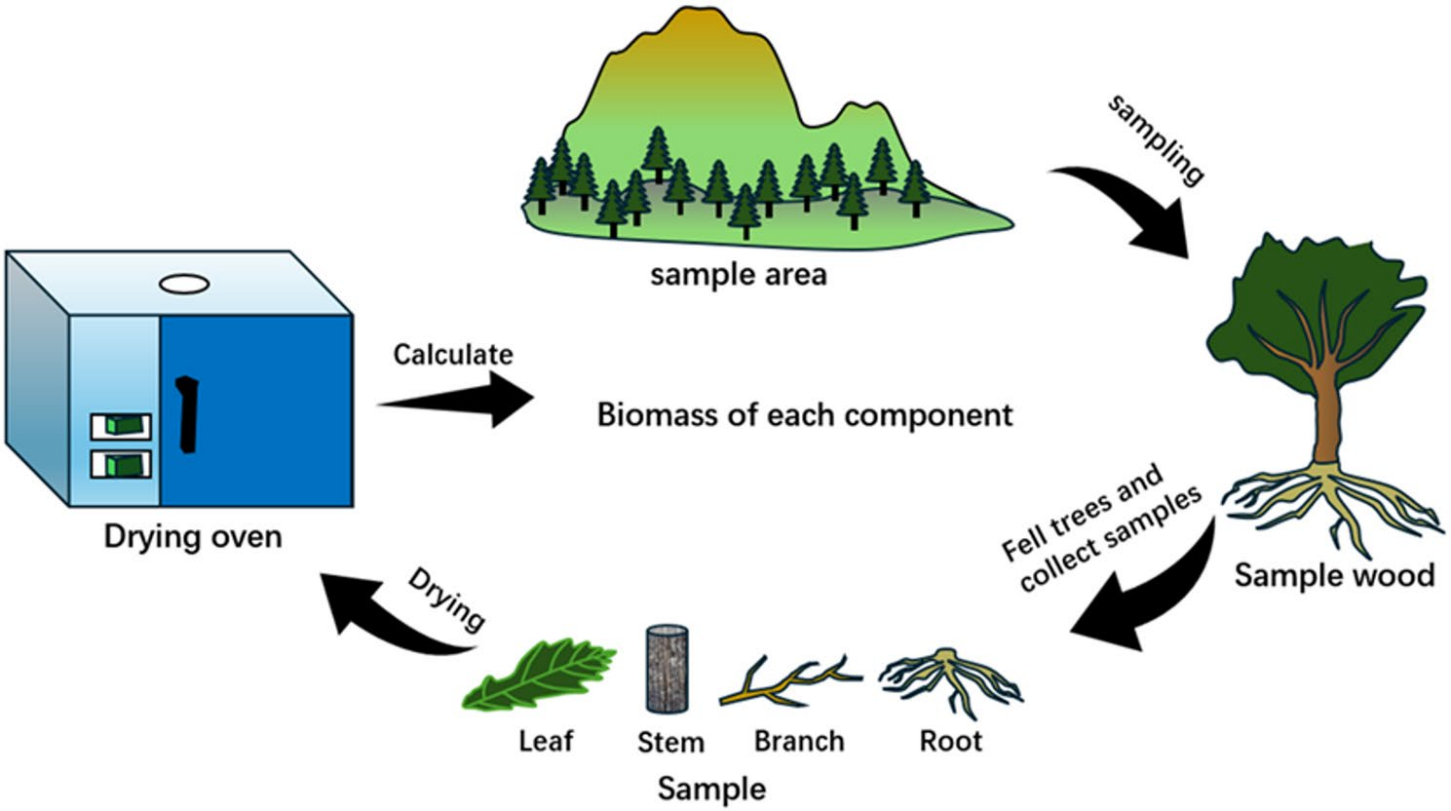
Flowchart of biomass collection for individual components of a Mongolian oak tree

After harvesting, each tree was divided into three layers, and the total fresh weight of branches was measured. The fresh weight of leafy and leafless branches was used to estimate the total branch and leaf biomass of each tree. Representative samples of branches and leaves were selected for fresh weight determination. The data acquisition process is shown in Fig. 2.

53 strains were selected from the total sample trees according to the diameter class for underground biomass determination. Dig out the whole root system, clean up the debris, and weigh the total fresh weight. Then the representative root samples were dried to constant weight at 85°C. The ratio of dry weight to fresh weight was used to calculate the biomass of aboveground and underground parts, and to determine the total biomass of trees. The carbon storage of each component of the individual tree was calculated by multiplying the biomass of each component specified in the national standard GB/T 43648 − 2024 ' Biomass model and carbon measurement parameters of main tree species ' (GB/T 43648 − 2024 2024) by its carbon content factor. The carbon content of stems was derived from the arithmetic mean of wood and bark carbon content. The total carbon storage on the ground is the sum of the carbon storage of each component (Carbon content factors: Stem: 0.4820, Branch: 0.4805, leaf: 0.4923). The basic statistics of the research data are shown in Table 1.

**Table 1:** Basic information statistics of research data

Variable	Mean	Max.	Min.	SD	CV
*DBH*/cm *H*/m Age Stem biomass/kg Foliage biomass/kg Branch biomass/kg Root biomass/kg Aboveground biomass/kg	15.51	33	1.5	9.75	0.63
	9.72 139 95.29 5.65 47.23 40.30 148.17	20.5 737.35 413.55 26.82 316.34 203.91 701.91	1.4 5 0.21 0.03 0.06 0.34 0.34	5.19 27.15 108.31 6.69 66.22 50.93 174.36	0.53 0.58 1.14 1.18 1.40 1.26 1.18

*Note* All the above are single plant data

This study mainly uses Excel and ArcMap 10.8 for data collation and mapping, and uses FORSTAT 3.0 and origin for parameter estimation and correlation analysis. ClimateAP is used to extract climate data, including 17 indicators: MAT (Mean Annual Temperature), MWMT (Mean Warmest Month Temperature), MCMT (Mean Coldest Month Temperature), TD (Temperature Difference), MAP (Mean Annual Precipitation), AHM (Annual Heat Moisture Index), DD < 0 (Degree-Days Below 0 °C), DD > 5 (Degree-Days Above 5 °C), DD < 18 (Degree-Days Below 18 °C), DD > 18 (Degree-Days Above 18 °C), NFFD (Number of Frost-Free Days), PAS (Precipitation as Snow), EMT (Extreme Minimum Temperature), EXT (Extreme Maximum Temperature), EREF (Hargreaves Reference Evapotranspiration), CMD (Hargreaves Climatic Moisture Deficit), RH (Relative Humidity).

### Research methods

#### Biomass allocation pattern of individual Mongolian oak

The biomass allocation patterns (including root biomass) of 53 individuals of Q. *mongolica* were observed with age, DBH and tree height as independent variables. Among them, the proportion of biomass of each component relative to DBH is expressed by the average proportion of biomass of each component among different diameter classes, which is divided into: 2,4,6,8,12,16,20,24,28 and 32 cm diameter classes. The proportion of biomass of each component relative to age was expressed by the average proportion of different age groups, which were 5–15,15–25,25–35,35–45,45–55,55–65,65–75,75–90,90–105 and 105–130 years, respectively. Similarly, the ratio of biomass of each component to tree height was expressed as the average ratio of different height groups, which was divided into: 2, 4, 6, 8, 10, 12, 14, 16, 18 and 20 m height classes.

#### Independent model

Before model construction, correlation analysis should be performed to examine the relationship between survey factors and to detect potential data errors. At present, the main model forms include linear model, logarithmic model, polynomial model and allometric growth model. Linear and polynomial models are subject to various restrictions in application, while logarithmic models require inverse transformation in the application process. Therefore, this study adopts a relatively intuitive allometric growth model. The allometric growth model can be further divided into: constant shape variation ratio (CAR) model and variable body shape variation ratio (VAR) model. The CAR equation has the characteristics of simple structure and stable parameters. Therefore, this study uses the CAR form to establish independent univariate and bivariate models for biomass and carbon storage components [19].

Biomass data often show heteroscedasticity, and heteroscedasticity needs to be eliminated in model parameter estimation. At present, the methods of eliminating heteroscedasticity mainly include logarithmic regression and weighted regression. Since logarithmic regression requires subsequent inverse transformation of the model, this study uses weighted regression to eliminate heteroscedasticity [5, 46, 49]. The weight function can be adjusted to 1/√(f(x)).

One-dimensional standing tree biomass and carbon storage model expression:

1 $$Y={a}_{0}\times\:{DBH}^{{a}_{1}}+\epsilon$$

Binary standing tree biomass and carbon storage model expression:

2 $$Y={a}_{0}\times\:{DBH}^{{a}_{1}}\times\:{H}^{{a}_{2}}+\epsilon$$

In the equations : $\:Y$ is biomass data (kg); DBH is diameter at breast height (cm); H is tree height (m) $\:{a}_{0}$, $\:{a}_{1}\:and$ $\:{a}_{2}$ are the parameter to be estimated.

#### Establishment of ground compatibility model

Traditional biomass prediction models often ignore the additivity due to the separate modeling of components, and the establishment of a compatible model with additivity can effectively solve this problem. During the growth and development of trees, the proportion of various biomass components shows a systematic change with the change of tree size, while the total proportion of all components remains constant at 1. The compatibility model mainly exists in two forms: the decomposition compatible biomass model and the aggregation compatible biomass model. In order to solve the compatibility problem, this study also developed a decomposed compatibility model and a aggregated compatibility model, and used the seemingly unrelated regression (SUR) method to estimate the model parameters.

3 $$\:\begin{array}{c}\left\{\begin{array}{c}{M}_{1}=\frac{1}{1+{g}_{1}\left(x\right)+{g}_{2}\left(x\right)}\times\:{f}_{1}\left(x\right)+\epsilon\:\\\:{M}_{2}=\frac{{g}_{1}\left(x\right)}{1+{g}_{1}\left(x\right)+{g}_{2}\left(x\right)}\times\:{f}_{1}\left(x\right)+\epsilon\:\\\:{M}_{3}=\frac{{g}_{2}\left(x\right)}{1+{g}_{1}\left(x\right)+{g}_{2}\left(x\right)}\times\:{f}_{1}\left(x\right)+\epsilon\:\end{array}\right.\end{array}$$

4 $$\:\begin{array}{l}\left\{\begin{array}{l}{M}_{1}={f}_{2}\left(x\right)+\epsilon\:\\\:{M}_{2}={f}_{3}\left(x\right)+\epsilon\:\\\:{M}_{3}={f}_{4}\left(x\right)+\epsilon\:\\\:{M}_{a}={f}_{2}\left(x\right)+{f}_{3}\left(x\right)+{f}_{4}\left(x\right)+\epsilon\:\end{array}\right.\end{array}$$

In the equations: *M*₁, *M*₂, *M*₃, and *M*_a_ represent the biomass of stems, leaves, branches, and aboveground biomass, respectively. *f*₁(x) denotes the estimated aboveground biomass derived from Eqs. (1) and (2). *f*₂(x), *f*₃(x), and *f*₄(x) are the aggregate fitted model expressions for stems, branches, and leaves, respectively, selected from independent models. *g*_i_(x) represents the proportional function of each organ (where *i* = 1, 2; 1 for branches and 2 for leaves), with expressions identical to Eqs. (1) and (2). To estimate carbon storage, biomass values can be multiplied by carbon content coefficients. By applying organ-specific carbon content factors to the biomass equations above, carbon storage values for each organ can be obtained. The compatible model formulation for carbon storage follows the same structure and is therefore not reiterated here. It is worth noting that there are two ways for the biomass model established in this paper to predict carbon storage. One is to directly bring the stand factor into the carbon storage compatibility model to obtain the above-ground carbon storage. If you want to obtain the carbon storage of a single plant, you need to calculate the underground carbon storage and add it. The second is that the biomass compatibility model is used to obtain the biomass of each organ, and then the carbon content is added to obtain the above-ground carbon storage, which is then substituted into the root-stem ratio model to calculate the underground carbon storage. However, it is not possible to directly add the biomass model of each organ to the carbon content per plant. This is because the data used in this paper is different from the national standard. In order to meet the two compatibility ideas of carbon storage, the carbon content per plant in the national standard cannot be directly substituted for calculation. Therefore, this paper also establishes a biomass model and a carbon storage model.

#### Aggregate biomass and carbon storage model with climate factors

Based on the model accuracy, the aggregate compatibility model is selected for the screening and inclusion of climate factors. The correlation analysis was used to screen the climate indicators, and the correlation table of each factor is shown in Table 2. According to the correlation between each component and related climatic factors, as well as the correlation between climatic factors themselves, DD < 0, PAS and TD were finally selected as the main climatic factors of stems, branches and leaves, respectively. Then these factors are incorporated into the compatibility model for further fitting.

5 $$\:\begin{array}{l}\left\{\begin{array}{l}{M}_{a}=\left({a}_{0}+{a}_{01}\times\:DD<0\right)\times\:{D}^{\left({a}_{1}+{a}_{11}*DD<0\right)}\times\:{H}^{\left({a}_{2}+{a}_{21}*DD<0\right)}\\\:\\\:+\left({b}_{0}+{b}_{01}\times\:PAS\right)\times\:{D}^{\left({b}_{01}+{b}_{11}*PAS\right)}\\\:+\left({f}_{0}+{f}_{01}\times\:TD\right)\times\:{D}^{\left({f}_{1}+{f}_{11}*TD\right)}+\epsilon\:\\\:{M}_{1}=\left({a}_{0}+{a}_{01}\times\:DD<0\right)\times\:{D}^{\left({a}_{1}+{a}_{11}*DD<0\right)} \\\: \times\:{H}^{\left({a}_{2}+{a}_{21}*DD<0\right)}+\epsilon\:\\\:{M}_{2}=\left({b}_{0}+{b}_{01}\times\:PAS\right)\times\:{D}^{\left({b}_{01}+{b}_{11}*PAS\right)}+\epsilon\:\\\:{M}_{3}=\left({f}_{0}+{f}_{01}\times\:TD\right)\times\:{D}^{\left({f}_{1}+{f}_{11}*TD\right)}+\epsilon\:\end{array}\right.\end{array}$$

#### Belowground-to-Aboveground biomass ratio model

The ratio of below-ground biomass to above-ground biomass (root-stem ratio) is the ratio of below-ground biomass to above-ground biomass. Due to the challenge of measuring belowground biomass, the number of sampled trees with complete biomass data (including roots) is limited. Of the 175 sample trees in this study, only about 1/3 of the sample trees were excavated for the determination of underground biomass. Therefore, a root-stem ratio model was established by using limited underground biomass samples and their corresponding aboveground biomass data. Then the ratio model is multiplied by the aboveground biomass model to estimate the underground biomass model. For the compatibility model, the corresponding multi-root-stem ratio model can also be used. This study uses the SUR method for joint parameter estimation. The simultaneous equations are as follows (the establishment method of the underground carbon storage root-stem ratio model is the same as this, and will not be repeated here).

Unitary root-stem ratio model expression:

6 $$\:\begin{array}{l}\left\{\begin{array}{l}{M}_{a}={a}_{0}\times\:{DBH}^{{a}_{1}}\\\:{M}_{b}=R\times\:{M}_{a}={b}_{0}\times\:{DBH}^{{b}_{1}}\times\:{M}_{a}\end{array}\right.\end{array}$$

Binary root-stem ratio model expression:

7 $$\:\begin{array}{l}\left\{\begin{array}{l}{M}_{a}={a}_{0}\times\:{DBH}^{{a}_{1}}\times\:{H}^{{a}_{2}}\\\:{M}_{b}=R\times\:{M}_{a}={b}_{0}\times\:{DBH}^{{b}_{1}}\times\:{H}^{{b}_{2}}\times\:{M}_{a}\end{array}\right.\end{array}$$

When using the root-stem ratio model, in order to reduce the error transfer between the models, the average estimation error can be calculated based on the correlation coefficient between underground biomass and aboveground biomass.

8 $$\:\begin{array}{c}{MPE}_{1}=\sqrt{1-\left(1-\frac{1}{K}\right)\times\:{r}^{2}}\times\:{MPE}_{2}\:\end{array}$$

In the equation: *K* is the ratio of the number of samples for aboveground biomass to belowground biomass, *r* is the correlation coefficient, *MPE₂* is the mean prediction error obtained using a limited sample size, MPE₁ is the mean prediction error of the compatible belowground biomass and carbon stock estimates after integrating a large-sample AGB model.

#### Model validation

There are different views on the evaluation and verification of the model. Most scholars believe that it is necessary to establish a separate validation data set for the test model, while Kozak et al. believed that it is not necessary to leave independent samples for cross-validation [20]. In order to make full use of the sample data, they recommend using the entire data set for model development. At present, the ‘Jackknife method’ is widely used as a verification method [38, 44]. Therefore, this study used all sample trees for model construction and used the following four evaluation indicators for evaluation: adjusted determination coefficient (R_adj_^2^), standard error of estimate (SEE), mean prediction error (MPE), and total relative error (TRE). Among them, MPE is the main evaluation index, and the above statistical indicators are calculated by the Jackknife method.

9 $$\:\begin{array}{c}{R}_{adj}^{2}=1-\frac{\left({1-R}^{2}\right)\times\:\left(N-1\right)}{N-P-1}\end{array}$$

10 $$\:\begin{array}{c}SEE=\sqrt{\frac{\sum\:_{i=1}^{N}{\left({Y}_{i}-\widehat{{Y}_{i}}\right)}^{2}}{\left(N-P\right)}\:}\end{array}$$

11 $$\:\begin{array}{c}TRE=\frac{\sum\:_{i=1}^{N}\left({Y}_{i}-\widehat{{Y}_{i}}\right)}{\sum\:_{i=1}^{N}\left(\widehat{{Y}_{i}}\right)}\times\:100\end{array}$$

12 $$\:\begin{array}{c}MPE={t}_{\alpha\:}\times\:\frac{\left(\frac{SEE}{\stackrel{-}{Y}}\right)}{\sqrt{N}}\times\:100\end{array}$$

In the equation: R² is the coefficient of determination of the formula, N is the number of sample units, P is the number of parameters, Y_i_ is the observed value of biomass or carbon stock, Ŷ_i_ is the model-estimated value of biomass or carbon stock, Ȳ is the arithmetic mean of the observed biomass or carbon stock values, tα is the t-value at confidence level α.

## Results

### Ccorrelations between biomass of different components and influencing factors

**Fig. 3:**
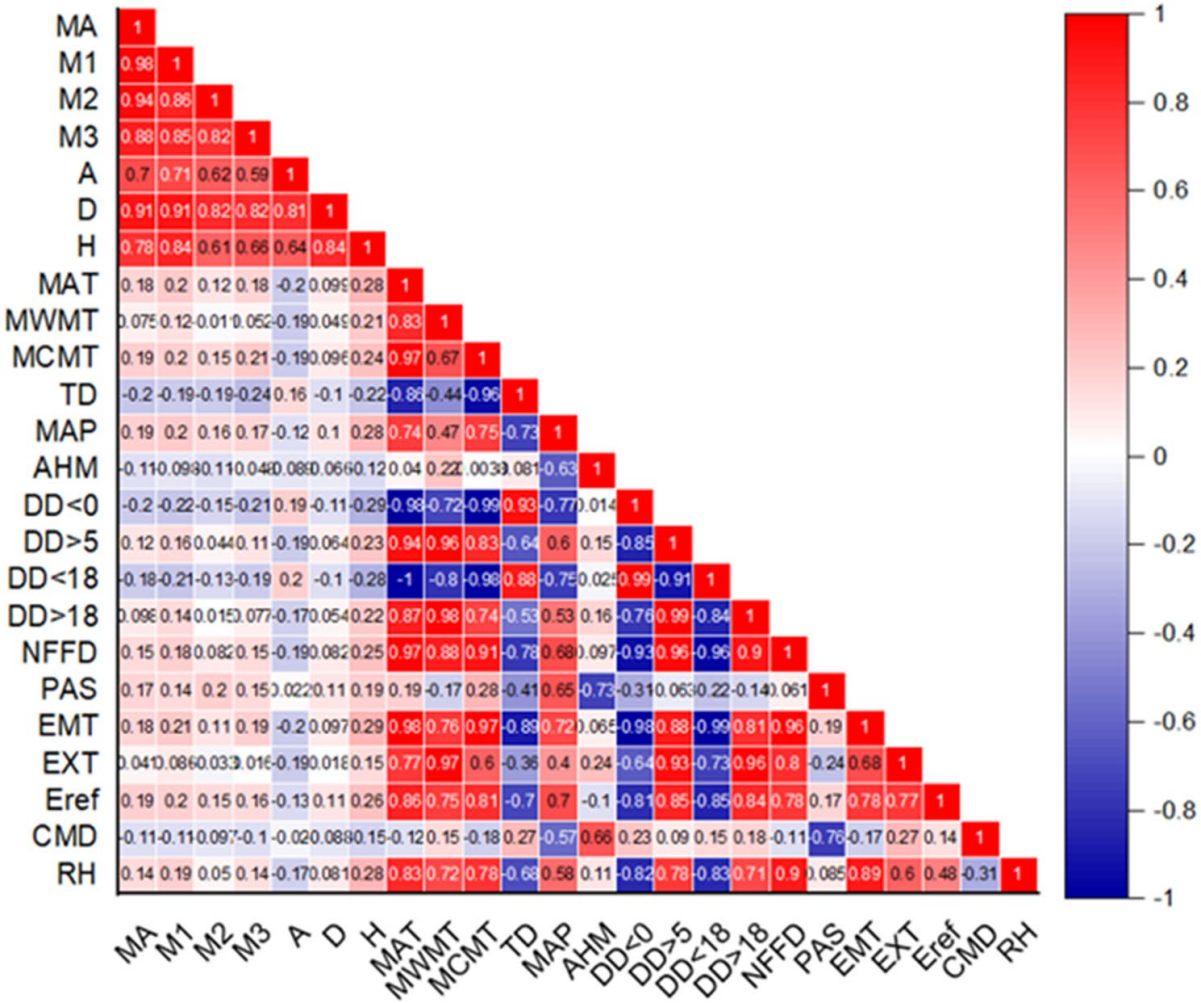
Correlations of biomass components with climate and stand factors

**Fig. 4:**
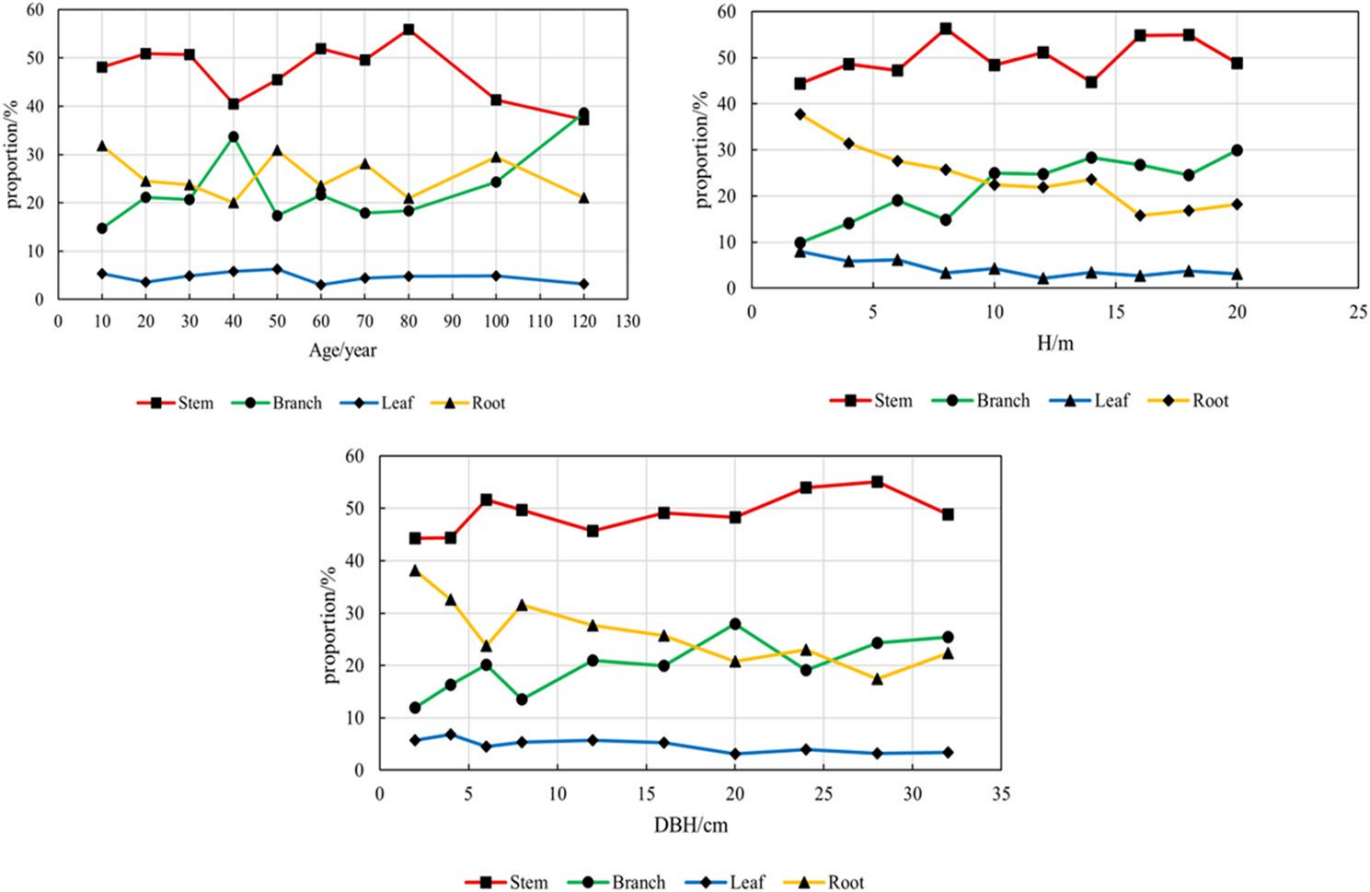
Changes in biomass allocation across stand factors

The correlation analysis between stand factors and biomass components showed that there were highly significant correlations among all stand factors (Fig. 3). Specifically, the correlation between DBH and all variables was more than 80%. The correlation between tree height and stem biomass was the strongest (84%), The correlation between tree height and leaf and branch biomass was relatively low (61%, 66%). The correlation between the biomass of each component was more than 80%. Among them, the correlation between stem, branch, leaf biomass and aboveground biomass was the strongest (98%, 94%, 88%). The correlation between forest factors and carbon storage of each component is similar to the observation results of biomass of each component. For climatic factors: the maximum correlation with biomass components was only 24%. The key climate-biomass relationships include: DD > 0 has the strongest correlation with stem biomass, PAS has the strongest correlation with branch biomass, and TD has the strongest correlation with leaf biomass (Fig. 4).

### The biomass allocation pattern of individual Mongolian oak trees

**Fig. 5:**
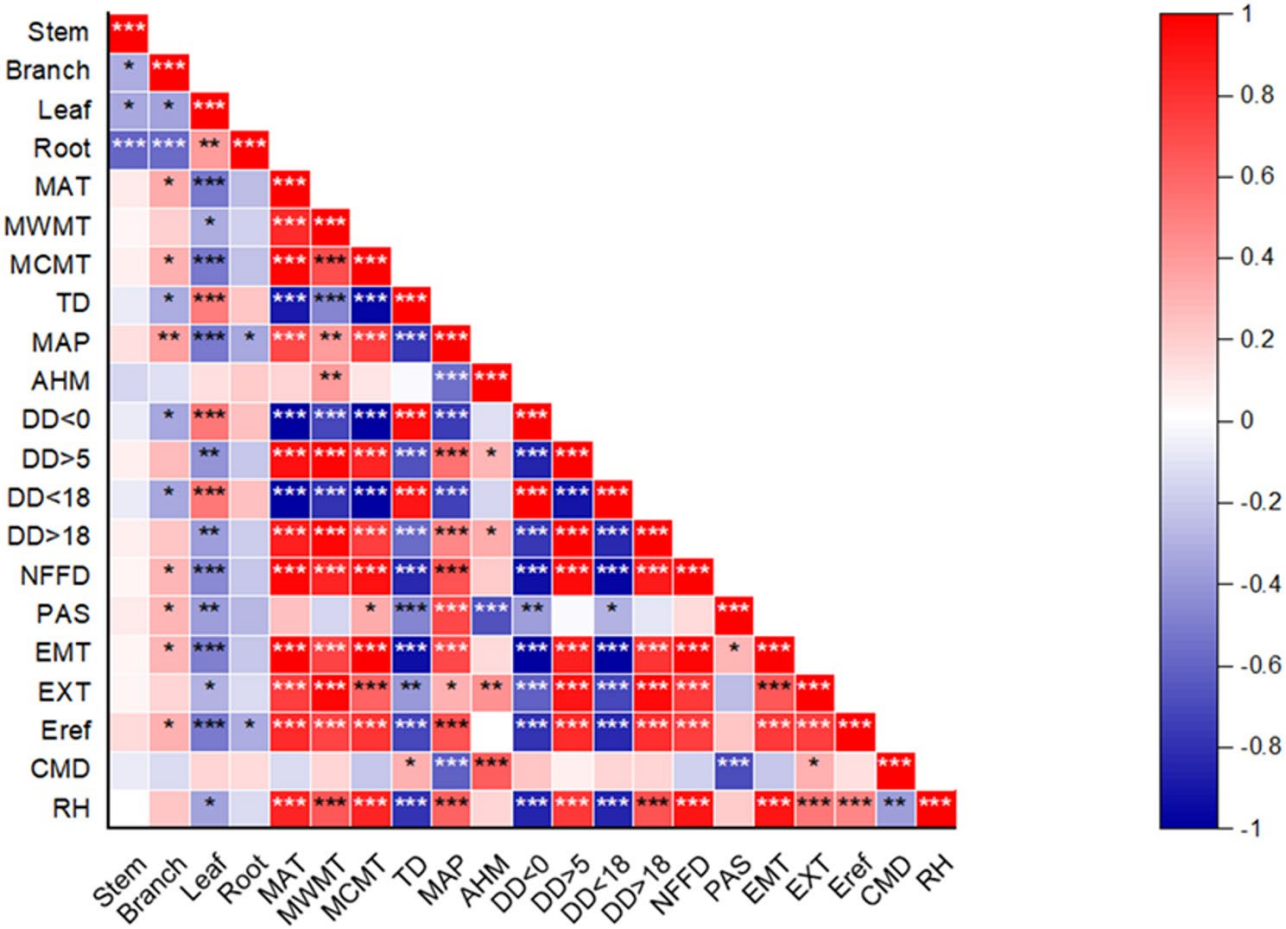
Correlations between biomass proportions of different components and climatic factors

According to the biomass distribution of Q. *mongolica*, the biomass of stem accounted for 27.13% ~ 75.28% of the total biomass (mean 49.12%), and the biomass of branches accounted for 7.56% ~ 45.38% of the total biomass (mean 20.20%). The leaf biomass was 0.95% −12.72% (mean 4.61%), and the root biomass was 9.92% −54.55% (mean 26.07%). The proportion of stems was the highest, the proportion of roots and branches was similar, and the proportion of leaves was the smallest. By age class: the proportion of stem biomass decreased with age, the proportion of root biomass decreased with age, the proportion of branch biomass increased with age, and the proportion of leaf biomass remained relatively stable among all age classes. By DBH class: Stem biomass proportion increases with diameter, Branch biomass proportion rises with diameter, Root biomass proportion decreases with diameter, Leaf biomass proportion shows minimal variation. Classification by tree height: the proportion of stem biomass increased with the increase of tree height, the proportion of branch biomass increased with the increase of tree height, the proportion of root biomass decreased with the increase of tree height, and the proportion of leaf biomass remained the same. In terms of the change of biomass proportion of different components with climate change, there was no significant correlation between stem biomass proportion and climate factors. The proportion of branch biomass was positively correlated with MAT, MCMT, NFFD, PAS, EXT, Eref and MAP, and negatively correlated with TD, DD < 0 and DD < 18. Leaf biomass ratio was significantly negatively correlated with MAT, MWMT, MCMT, MAP, DD > 5, DD > 18, NFFD, PAS, EMT, EXT, Eref and RH, and significantly positively correlated with TD, DD < 0 and DD < 18. The proportion of root biomass was significantly negatively correlated with MAP and Eref. Figure 5 shows the correlation between the biomass ratio of different components and climatic factors (Fig. 5).

### Independent biomass and carbon storage models of individual tree components

For the aboveground biomass of Q. *mongolica*, the *R*_*adj*_^*2*^ of the unary and binary models ranged from 0.7048 to 0.9618, the *TRE* was within ± 1%, and the MPE did not exceed the 10% threshold required for model accuracy (Table 2). The main findings include: For aboveground biomass and stem biomass, the bivariate model is superior to the univariate model. For branch biomass and leaf biomass, univariate models showed better performance than bivariate models. The underground biomass model showed slightly lower accuracy, and the univariate model performed better than the bivariate model.

**Fig. 6:**
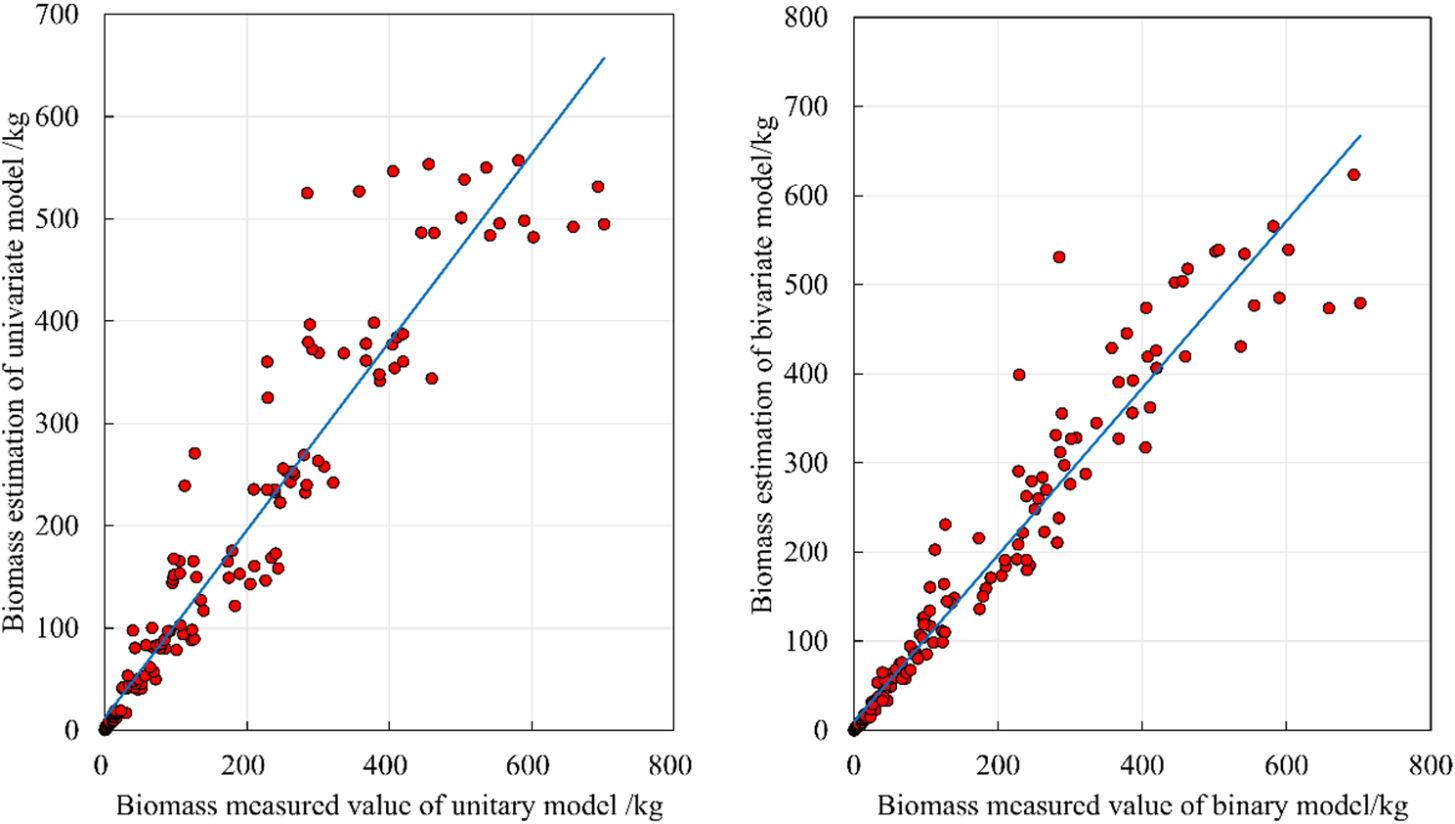
Comparison of estimation results of one-dimensional and two-dimensional aboveground biomass models

Consequently, in the aggregated model. Stem biomass was fitted using a bivariate model form. Branch and leaf biomass were fitted using univariate model forms. The comparative performance of biomass models is illustrated in Fig. 6. The carbon stock models showed comparable evaluation metrics to the biomass models, albeit with slight parameter variations (Table 3).

**Table 2:** Parameter Estimation and evaluation index of biomass independent model

Item	Model structure	a_0_	a_1_	a_2_	*R* ^2^_adj_	SEE/kg	TRE/%	MPE/%
Aboveground biomass	*M*_*a*_ *=a*_*0*_ *D ^*a1*^	0.0956	2.4803		0.9134	51.32	−0.01	5.17
	*M*_*a*_ *=a*_*0*_ *D ^*a1*^ * H ^*a2*^	0.0588	2.1205	0.6270	0.9375	43.58	−0.01	4.39
Stem biomass	*M*_*1*_ *= a*_0_*D^*a1*^	0.1057	2.3113		0.8824	37.14	−0.03	5.82
	*M*_*1*_ *= a*_0_*D^a1^* H^*a2*^	0.0445	1.7307	1.0389	0.9618	21.17	0.01	3.31
Branch biomass	*M*_*2*_ *= a*_0_*D^*a1*^	0.0069	2.9374		0.8028	29.41	0.04	9.29
	*M*_*2*_ *= a*_0_*D^a1^* H^*a2*^	0.0076	3.0193	−0.1361	07988	29.70	−0.05	9.38
Leaf biomass Belowground biomass	*M*_*3*_ *= a*_0_*D^*a1*^	0.0162	2.0129		0.7115	3.59	0.02	9.49
	*M*_*3*_ *= a*_0_*D^a1^* H^*a2*^ *M*_*b*_ *=a*_*0*_ *D ^*a1*^ *M*_*b*_ *=a*_*0*_ *D ^*a1*^ * H ^*a2*^	0.0168 0.0523 0.0496	1.9820 2.2585 2.1323	0.0225 0.1824	0.7048 0.7887 0.7839	3.63 23.41 23.67	−0.10 0.09 −0.27	9.60 16.02 16.21

**Table 3:** Parameter Estimation and evaluation of independent model of carbon storage

Item	Model structure	a_0_	a_1_	a_2_	*R* ^2^_adj_	SEE/kg	TRE/%	MPE/%
Aboveground carbon storage	*C*_*a*_ *=a*_*0*_ *D ^*a1*^	0.0462	2.4795		0.9134	24.72	−0.01	5.17
	*C*_*a*_ *=a*_*0*_ *D ^*a1*^ * H ^*a2*^	0.0284	2.1195	0.6272	0.9376	20.98	−0.01	4.39
Stem carbon storage	*C*_*1*_ *= a*_0_*D^*a1*^	0.0509	2.3113		0.8824	17.90	−0.03	5.82
	*C*_*1*_ *= a*_0_*D^a1^* H^*a2*^	0.0214	1.7307	1.0389	0.9618	10.20	0.01	3.31
Branch carbon storage	*C*_*2*_ *= a*_0_*D^*a1*^	0.0033	2.9374		0.8028	14.13	0.04	9.29
	*C*_*2*_ *= a*_0_*D^a1^* H^*a2*^	0.0037	3.0193	−0.1361	07988	14.27	−0.05	9.38
Leaf carbon storage Belowground carbon storage	*C*_*3*_ *= a*_0_*D^*a1*^	0.0080	2.0129		0.7115	1.77	0.02	9.49
	*C*_*3*_ *= a*_0_*D^a1^* H^*a2*^ *C*_*b*_ *=a*_*0*_ *D ^*a1*^ *C*_*b*_ *=a*_*0*_ *D ^*a1*^ * H ^*a2*^	0.0083 0.0245 0.0228	1.9820 2.2585 2.2801	0.0225 0.1859	0.7048 0.7887 0.7839	1.79 10.95 11.07	−0.10 0.09 −0.26	9.60 16.02 16.21

### Decomposition compatible aboveground biomass and carbon storage model

The *R*_*adj*_^*2*^ of the univariate and bivariate additive biomass models was between 0.7049 and 0.9604, *TRE* did not exceed ± 1%, and *MPE* remained below 10% (Table 4). These results indicate that the decomposition model successfully solves the problem of ensuring that the sum of biomass of each component is equal to the total aboveground biomass. The parameters and evaluation indexes of the carbon storage decomposition compatibility model and the biomass decomposition compatibility model are similar (Table 5).

**Fig. 7:**
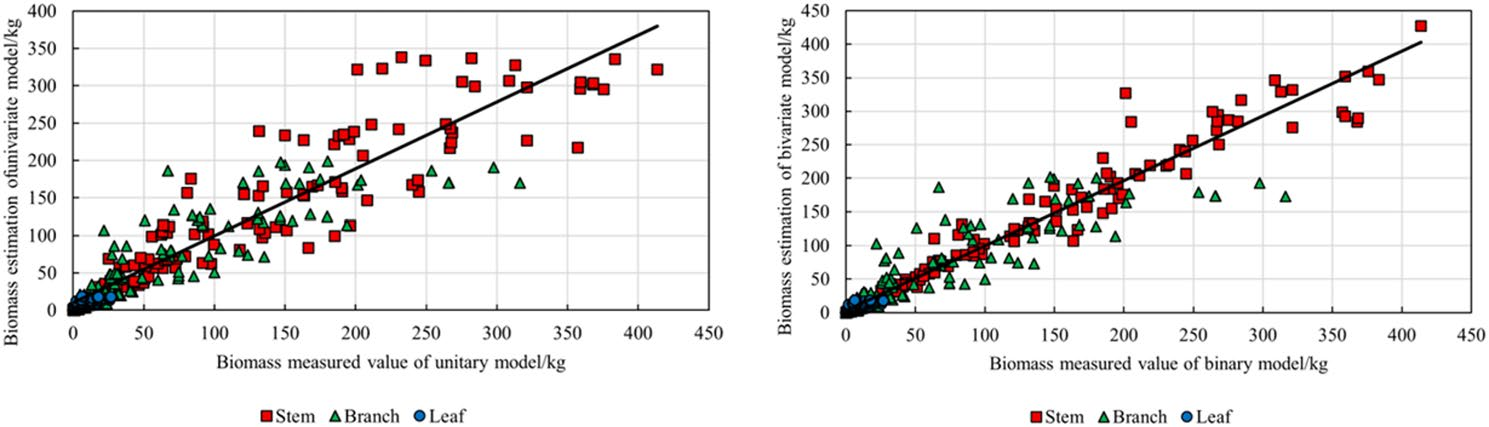
Comparison between measured and estimated biomass values from univariate and bivariate decomposition-compatible models

Consistent with the performance of the independent models, the bivariate models for aboveground biomass and stem biomass outperformed the univariate models, whereas the univariate models for branch biomass and leaf biomass were superior to their bivariate counterparts. However, the additive models exhibited slightly lower accuracy compared to the independent models. A comparison of model fitting is illustrated in Fig. 7.

**Table 4:** Parameter estimation and evaluation index of decomposition aboveground biomass compatibility model

Item	Model structure	a_0_	a_1_	a_2_	*R* ^2^_adj_	SEE/kg	TRE%	MPE%
Stem biomass	*M*_*1*_ *= a*_0_*D^*a1*^				0.8812	37.34	0.01	5.85
	*M*_*1*_ *= a*_0_*D^a1^*H^*a2*^				0.9604	21.56	0.02	3.38
Branch biomass	*M*_*2*_ *= a*_0_*D^*a1*^	0.0686	0.6133		0.8007	29.56	−0.04	9.34
	M_*2*_ = *a*_0_*D^a1^*H^*a2*^	0.1816	1.3035	−1.2162	0.7968	29.85	−0.07	9.43
Leaf biomass	M_*3*_ = a_0_*D^a1^	0.1646	−0.3209		0.7075	3.62	0.03	9.56
	M_*3*_ = a_0_*D^a1^*H^a2^	0.3426	0.2810	−1.0139	0.7049	3.63	−0.08	9.60

**Table 5:** Parameter estimation and evaluation of aboveground carbon storage compatibility model

Item	Model structure	a_0_	a_1_	a_2_	*R* ^2^_adj_	SEE/kg	TRE%	MPE%
Stem carbon storage	*C*_*1*_ *= a*_0_*D^*a1*^				0.8812	18.00	0.01	5.85
	*C*_*1*_ *= a*_0_*D^a1^*H^*a2*^				0.9604	10.39	0.02	3.38
Branch carbon storage	*C*_*2*_ *= a*_0_*D^*a1*^	0.0684	0.6133		0.8007	14.20	−0.04	9.34
	*C*_*2*_ *= a*_0_*D^a1^*H^*a2*^	0.1810	1.3037	−1.2163	0.7968	14.34	−0.07	9.43
Leaf carbon storage	*C*_*3*_ *= a*_0_*D^*a1*^	0.1680	−0.3207		0.7075	1.78	0.03	9.56
	*C*_*3*_ *= a*_0_*D^a1^*H^*a2*^	0.3502	0.2812	−1.0145	0.7049	1.79	−0.08	9.60

### Aggregative aboveground biomass and carbon stock compatible models

**Fig. 8:**
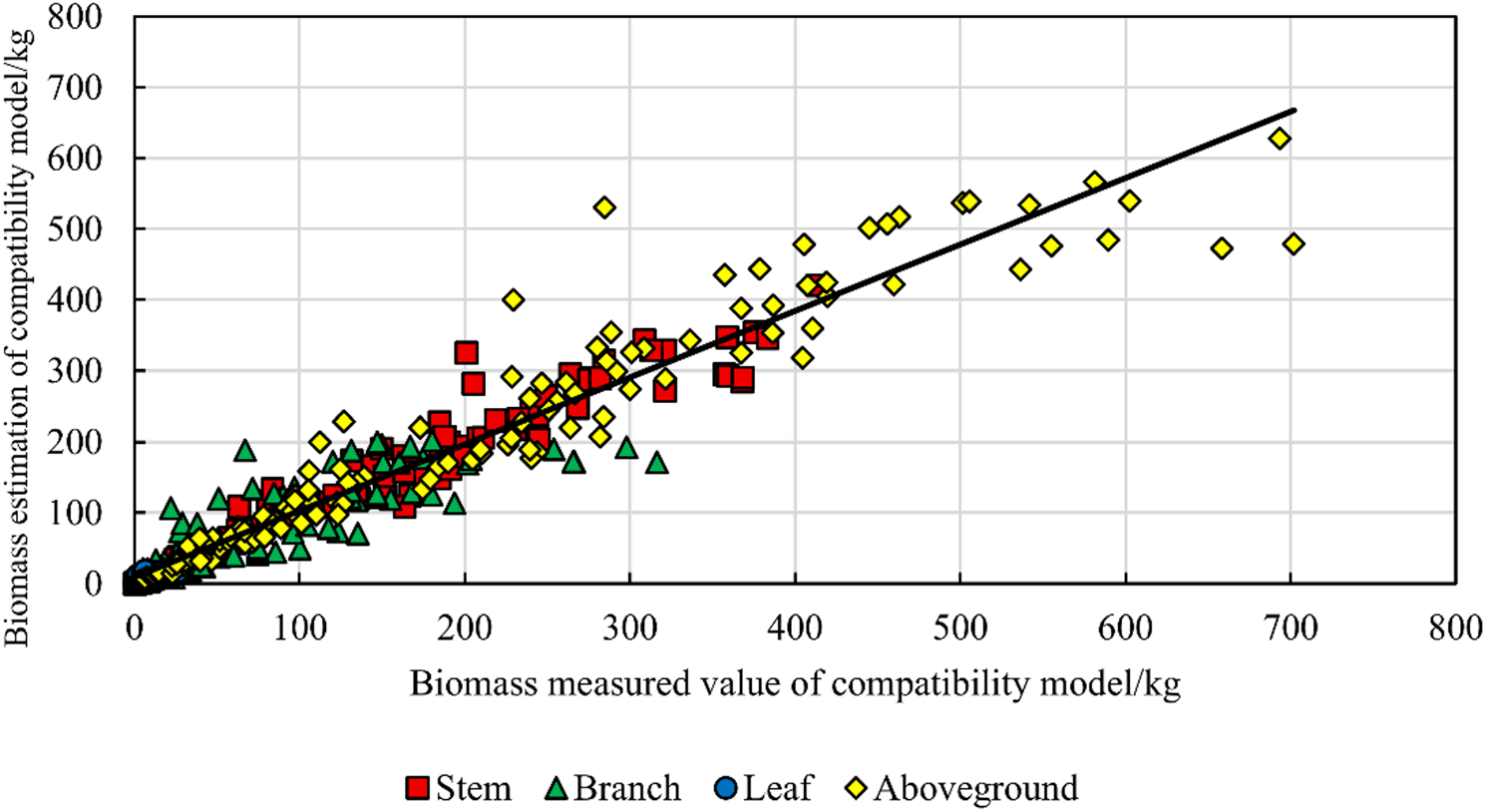
Comparison of measured versus estimated biomass values from the aggregation-compatible model

The R_adj_^2^ of the model is between 0.7113 and 0.9671, TRE is less than 1%, and MPE is kept below 10% (Table 6). These results indicate that the aggregate compatibility model can be effectively applied to the prediction of individual tree biomass of Q. *mongolica* in Northeast China. By comparing the aggregated compatibility model (based on algebraic summation) with the decomposed compatibility model (under total biomass control), it was found that the aggregated compatibility model had higher prediction accuracy in stem biomass, branch biomass and leaf biomass. For aboveground biomass, the accuracy of the aggregated compatibility model is slightly lower than that of the bivariate independent model, but it is still better than the univariate independent model. The parameters and evaluation indexes of the carbon storage aggregation compatibility model and the biomass aggregation compatibility model are similar (Table 7). The comparison of model fitting is shown in Fig. 8.

**Table 6:** Parameter estimation and evaluation index of aggregate aboveground biomass compatibility model

Item	a_1_	a_2_	a_3_	*R* ^2^_adj_	SEE/kg	TRE%	MPE%
Stem biomass/kg Branch biomass/kg Leaf biomass/kg Aboveground biomass/kg	0.0458 0.0068 0.0168	1.7481 2.9438 2.0005	1.0067	0.9617 0.8025 0.7113 0.9361	21.20 29.43 3.59 44.07	0.03 −0.04 −0.07 0.00	3.32 9.30 9.49 4.44

*Note* In order to avoid parameter confusion, *a*_*1*_,* a*_*2*_ and *a*_*3*_ refer to the first, second and third parameters of each function expression

**Table 7:** Parameter Estimation and evaluation index of aggregate aboveground carbon storage compatibility model

Item	a_1_	a_2_	a_3_	*R* ^2^_adj_	SEE/kg	TRE%	MPE%
Stem carbon storage/kg Branch carbon storage/kg Leaf carbon storage/kg Aboveground carbon storage/kg	0.0220 0.0033 0.0081	1.7483 2.9436 2.0083	1.007 1	0.9617 0.8025 0.7106 0.9362	10.22 14.14 1.77 21.22	0.03 −0.04 −0.13 0.00	3.32 9.30 9.51 4.43

*Note* In order to avoid parameter confusion, *a*_*1*_,*a*_*2*_ and *a*_*3*_ refer to the first, second and third parameters of each function expression

### Aggregate biomass and carbon storage model with climate factors

**Fig. 9:**
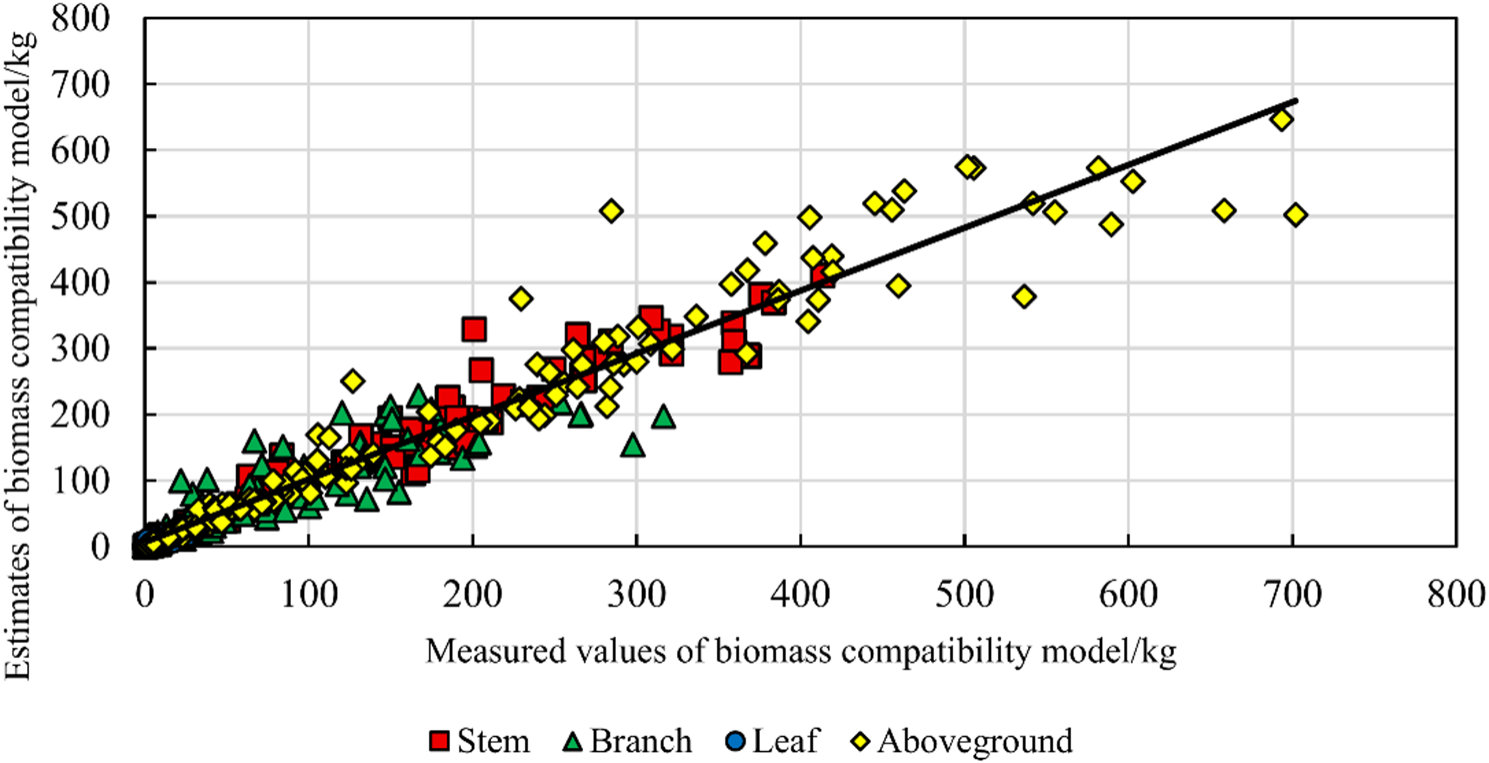
Comparison of measured versus estimated biomass values from the climate-factor-incorporated aggregation-compatible model

After incorporating climate indicators into the independent models of stem, branch and leaf biomass in the aggregated compatibility model for re-fitting, the accuracy of the fitted climate-compatible aggregation model was significantly improved (Table 8). In terms of MPE, the accuracy of the aboveground biomass model was increased by 0.15, and the accuracy of the stem biomass model was slightly reduced by 0.01. The accuracy of the branch biomass model was improved by 0.48. The accuracy of the leaf biomass model was improved by 1.39. In general, except for a slight decrease in the accuracy of stem biomass, the overall accuracy of the compatibility model showed a greater enhancement. This small decrease in the stem biomass model may be due to the increase in complexity caused by too many model parameters. The parameters of the aggregation-compatible biomass and carbon storage model including climate factors are listed in Table 8, and the evaluation indicators are listed in Table 9. The measured biomass-estimated biomass of the aggregated compatibility model considering climatic factors is shown in Fig. 9.

**Table 8:** Parameter estimates of the aboveground aggregation compatible biomass model incorporating Climatic factors

Item	a_0_	a_01_	a_1_	a_11_	a_2_	a_21_
Aboveground biomass Aboveground carbon storage						
Stem biomass	0.042623	0.000005	1.613553	0.000116	1.259482	−0.000230
Stem carbon storage	0.022058	0.000002	1.638036	0.000101	1.204234	−0.000195
Branch biomass	−0.003729	0.000210	3.163793	−0.004557		
Branch carbon storage	−0.001795	0.000100	3.167026	−0.004585		
Leaf biomass	−0.109317	0.003615	4.483994	−0.069648		
Leaf carbon storage	−0.060362	0.001944	4.789116	−0.077394		

**Table 9:** Evaluation metrics of the aboveground aggregation-compatible biomass model incorporating Climatic factors

Item	*R* ^2^_adj_	SEE/kg	TRE/%	MPE/%
Aboveground biomass	0.9403	42.62	0.01	4.29
Stem biomass	0.9615	21.26	0.13	3.33
Branch biomass	0.8223	27.92	−0.17	8.82
Leaf biomass	0.7888	3.07	−0.45	8.12
Aboveground carbon storage	0.9403	20.53	0.04	4.29
Stem carbon storage	0.9615	10.24	0.19	3.33
Branch carbon storage	0.8225	13.41	−0.21	8.81
Leaf carbon storage	0.7913	1.50	−0.45	8.07

### Compatible underground biomass root-stem ratio models and carbon stock models

After the accuracy of the underground biomass model is adjusted by the correlation coefficient, R_adj_^2^ is between 0.7702 and 0.7801, TRE is not more than 1%, and MPE is kept below 15%, which meets the accuracy requirements of the model. These results indicate that these models successfully solve the compatibility problem between aboveground and belowground biomass and carbon storage estimation, making them suitable for predicting belowground biomass and carbon storage. In addition, the assessment indicators of underground biomass and carbon storage did not change much, and the univariate compatibility model showed higher accuracy than the bivariate compatibility model (Table 10).

**Table 10:** Estimation and evaluation of the parameters of the underground biomass and carbon storage compatibility model

Model structure	b_1_	b_2_	b_3_	*R* ^2^_adj_	SEE/kg	TRE/%	MPE/%
*M*_*b*_ *=M*_*a*_ *b_*1*_ *D ^*b2*^	0.6926	−0.2848		0.7801	23.88	0.09	10.97
*M*_*b*_ *=M*_*a*_ *b_*0*_ *D ^*b1*^ *H ^*b2*^	0.8966	0.013 3	−0.4805	0.7702	24.42	−0.27	11.22
*C*_*b*_ *=M*_*a*_ *b_*0*_ *D ^*b1*^	0.6724	−0.2845		0.7801	11.17	0.09	10.97
*C*_*b*_ *=M*_*a*_ *b_*0*_ *D ^*b1*^ * H ^*b2*^	0.8707	0.0139	−0.4809	0.7702	11.42	−0.27	11.22

When using the aggregate compatibility model to calculate the underground biomass of a single tree, the aboveground biomass calculated under the aggregate compatibility model can be multiplied by the unary root-stem ratio model to obtain the underground biomass. Because the correlation coefficient of the root-stem ratio model is the same, the evaluation index of the compatibility model is the same as that of the unary model.

## Discussion

The proportion of single tree biomass allocation will be different due to different species [27]. In this study, the biomass allocation model of Q. *mongolica* was analyzed. The results showed that the biomass allocation proportion of branches increased with the change of stand level factors, which was consistent with the previous research results [17]. This is because the crown of Q. *mongolica* is large and oval with extensive branches, resulting in no obvious differentiation between stem and branch, resulting in a gradual increase in the proportion of branch biomass. The leaves of Q. *mongolica* are single, and the edges are wavy, so the proportion of leaf biomass remains relatively stable. With the proliferation of branches, leaf biomass increased, but due to leaf characteristics, the proportion was always small and stable. On the contrary, the proportion of root biomass decreased with the increase of stand factors, which was consistent with the previous research results [35, 39, 45]. Therefore, the accurate measurement of branch biomass will significantly affect the accuracy of the model for estimating the biomass and carbon storage of large-diameter and old-aged Q.*mongolica* forests with large tree height. Through correlation analysis, it can be seen that the correlation between tree height and stem biomass is the highest, followed by total aboveground biomass and root biomass, while the correlation with leaf biomass and branch biomass is relatively low, which is also consistent with the model fitting results.

From the perspective of the influence of climatic factors on biomass allocation, the influence of climate change on the stem of Q. *mongolica* was relatively small. MAT, MAP and PAS were significantly positively correlated with the proportion of branch biomass (the correlation between each climate factor and individual tree biomass does not exceed 30%). This may be due to the extension of the growing season under higher annual temperature, precipitation and snowfall conditions, which promotes the development of branches. Adequate water supply may reduce growth constraints, allowing more resources to be allocated to shoots after root needs are met, while increased snowfall may protect the base of shoots from extreme low temperatures. This is also consistent with previous studies [36, 55]. TD, DD < 0 and DD < 18 were significantly positively correlated with leaf biomass ratio. Under larger annual temperature fluctuations and more low temperature days, Q.*mongolica* may respond to low temperature stress by increasing leaf biomass for photosynthetic compensation. Large annual temperature difference may also lead to leaf thickening, This is also in line with previous biological analysis of Q.*mongolica* [9, 23]. The significant negative correlation between MAP and root biomass allocation ratio may reflect that under sufficient rainfall conditions, plants preferentially allocate resources to aboveground parts, thereby reducing the allocation of root biomass [47].

The results show that compared with the decomposition compatibility model, the independent model shows higher accuracy, while the aggregation compatibility model has slightly higher accuracy than the independent model. This difference is due to the parameter instability caused by many parameters and variables contained in the simultaneous equation system. Nevertheless, the compatibility model ensures the additivity of biomass and carbon storage between individual trees, as well as the compatibility between aboveground and belowground biomass components. In this study, through correlation analysis, DD < 0, PAS and TD were selected as the parameters of the independent stem biomass model, branch biomass model and leaf biomass model in the aggregate compatibility system, respectively. Subsequently, a comprehensive compatible biomass model containing climatic factors was obtained by re-fitting, and its accuracy was better than the original compatible model. In view of the common heteroscedasticity in biomass data, this study used weighted regression for model fitting. The TRE remained within ± 1%, indicating that the weighted allometric growth equation used in this study can effectively fit the single tree biomass model and carbon storage model. The introduction of climate factors into the independent biomass model of each component used in this paper not only improves the accuracy of the total aboveground biomass model, but also enhances the accuracy of the respective models of each component. The method used in this paper can provide a theoretical basis for the biomass and carbon storage compatibility model of other tree species.

After incorporating tree height as a variable, the bivariate model showed higher accuracy than the univariate model in estimating aboveground biomass, carbon storage and stem biomass and carbon storage, which was consistent with previous research results [13, 46] . However, for branch biomass, leaf biomass, carbon storage and belowground biomass and carbon storage, the accuracy of the univariate model was slightly better than that of the bivariate model, which was consistent with previous studies [26, 51]. In terms of overall accuracy, the stem biomass and carbon storage model have the highest accuracy, followed by the branch biomass and carbon storage model, and the leaf biomass and carbon storage model have the lowest accuracy. This trend is consistent with the results of Wang et al. [38]. The low estimation accuracy of the underground biomass model may be related to the developed root system, strong tillering ability and operational errors in the sampling process. In this study, the Jackknife method was used to verify the model, and all the evaluation indexes met the accuracy standard, which confirmed the applicability of the model in the estimation of biomass and carbon storage of Q. *mongolica* in Northeast China.

After model selection, the aboveground biomass compatibility model finally selected the aggregated compatibility model as the basic model. The climate factors with the highest correlation between the selected basic model and each biomass component were re-fitted as parameters. The results showed that the addition of these climate factors significantly improved the model accuracy of branch and leaf biomass, indicating that the addition of climate factors can effectively improve the model accuracy, which is consistent with previous research results [10, 14]. Among the climatic factors, the negative correlation between stem biomass and DD < 0 was the strongest. This relationship may be attributed to a decrease in physiological activity (e.g., photosynthesis) caused by a decrease in stomatal enzyme activity induced by low temperature. In addition, the shortening of the growing season in the negative accumulated temperature area also limited the duration of stem biomass accumulation. The positive correlation between branch biomass and snowfall was the strongest. This pattern is likely to reflect the strategy of Q. *mongolica* to allocate more resources to branch extension in snowy years to take advantage of abundant spring water and compete for canopy space. On the contrary, the negative correlation between branch biomass and annual temperature change was the strongest. This inverse relationship may be due to the compression of the growing season caused by temperature fluctuations and the direct physiological stress of extreme temperatures on leaves.

In this study, the analysis of biomass allocation patterns of Q. *mongolica* is helpful to better understand the formation and change mechanism of single tree biomass of Q. *mongolica*, so as to provide a theoretical basis for more accurate management of single tree growth, biomass estimation model establishment and carbon storage measurement of Q. *mongolica*., Compared with the previous Q.*mongolica* single tree model, this study has more comprehensive data and a wider range of research areas, and the biomass and carbon storage compatibility model with climate factors established in this paper has significantly improved the accuracy of branch biomass and leaf biomass models, indicating that it is necessary to add climate factors to the model [42, 43]. In field work, climate data can be obtained by measuring DBH, tree height and individual tree position information to predict the biomass and carbon storage of each component of Q. *mongolica*.

The development of individual tree biomass and carbon storage models of Q. *mongolica* can benefit from more diverse approaches. At present, deep learning techniques, such as random forests, artificial neural networks, support vector regression, and gradient boosting regression trees, have shown promising capabilities in building accurate prediction models using limited sample data, while greatly reducing the need for destructive felling of trees [25, 30, 31, 37]. Adding additional forest factor variables, such as crown width and length, can also improve the accuracy of the model [32]. At the same time, more factors can also improve the accuracy of the model. For example, Wang et al. used the comprehensive competition index Hegyi ‘s CI and relative diameter Rd for model fitting [40]. Liu et al. fitted the site condition factor as a dummy variable to improve the accuracy of the model [22]. Trying to establish a mixed effect model in the method can also greatly improve the accuracy of the model [56]. The use of grading methods such as data stratification according to site quality and age can also improve the accuracy of the model [21]. Since the individual tree models established in this study are all based on trees with DBH less than 33 cm and tree height less than 20.5 m, all the models established are only applicable to the prediction of individual tree biomass and carbon storage with DBH less than 33 cm and tree height less than 20.5 m, while the univariate compatible univariate model is only applicable to individual trees with DBH less than 33 cm.

## Conclusion

In this study, the biomass allocation pattern of Q.*mongolica* was analyzed by using the data of biomass, tree height, DBH and age of 175 Q.*mongolica* individuals in Northeast China. The results showed that the biomass allocation had different trends: the proportion of belowground biomass decreased with the increase of tree height, DBH and age. The proportion of branch biomass increased with the increase of tree height, DBH and age. Therefore, when estimating the biomass and carbon storage of large-diameter Mongolian oak trees, the accurate measurement of branch biomass becomes particularly important for model accuracy.

The comparison between univariate and bivariate biomass and carbon storage models showed that the bivariate model was superior to the univariate model for aboveground and stem components, while the univariate model was superior to the bivariate model for leaf and branch components. This indicated that the introduction of tree height significantly improved the estimation accuracy of stem biomass and carbon storage of Q.*mongolica*, but had little effect on branch and leaf components. Both models can be used to estimate the biomass and carbon storage of Q. *mongolica*. Compared with the decomposed compatibility model, the aggregated compatibility model showed higher model accuracy for individual Q. *mongolica*.

By incorporating the most relevant climatic factors into the independent models of each component, the selected polymeric compatibility model was re-fitted. The evaluation indicators showed that the accuracy of the total aboveground biomass, branch biomass and leaf biomass models was improved after the climate factors were included, while the performance of the stem biomass model was slightly reduced, which may be due to the over-fitting of the model. The established decomposition-based aboveground compatibility model, aggregation-based aboveground compatibility model and rhizome ratio model with climatic factors can provide a reliable method for estimating the biomass and carbon storage of all components on the individual tree scale of Q.*mongolica.*

## Data Availability

No datasets were generated or analysed during the current study.
